# Role of Mitochondria in Parvovirus Pathology

**DOI:** 10.1371/journal.pone.0086124

**Published:** 2014-01-21

**Authors:** Jonna Nykky, Matti Vuento, Leona Gilbert

**Affiliations:** Department of Biological and Environmental Science, and Nanoscience Center, University of Jyväskylä, Jyväskylä, Finland; University of Pecs Medical School, Hungary

## Abstract

Proper functioning of the mitochondria is crucial for the survival of the cell. Viruses are able to interfere with mitochondrial functions as they infect the host cell. Parvoviruses are known to induce apoptosis in infected cells, but the role of the mitochondria in parvovirus induced cytopathy is only partially known. Here we demonstrate with confocal and electron microscopy that canine parvovirus (CPV) associated with the mitochondrial outer membrane from the onset of infection. During viral entry a transient depolarization of the mitochondrial transmembrane potential and increase in ROS level was detected. Subsequently, mitochondrial homeostasis was normalized shortly, as detected by repolarization of the mitochondrial membrane and decrease of ROS. Indeed, activation of cell survival signalling through ERK1/2 cascade was observed early in CPV infected cells. At 12 hours post infection, concurrent with the expression of viral non-structural protein 1, damage to the mitochondrial structure and depolarization of its membrane were apparent. Results of this study provide additional insight of parvovirus pathology and also more general information of virus-mitochondria association.

## Introduction

Mitochondria are important organelles for the cell as they produce energy, regulate redox balance and maintain Ca^2+^ homeostasis. In cell signalling the mitochondria regulate cell responses to different cellular situations determining the fate of cell from survival to death [1]–[3]. In viral infections, mitochondria have a role in innate immunity by activating interferon production [4]. Mitochondrial dysfunction is associated with numerous diseases such as neurodegenerative diseases, diabetes and cancer [5]–[11]. Among the factors leading to mitochondrial dysfunction are depolarization of the mitochondrial transmembrane potential (ΔΨm), changes in expression of mitochondrial proteins and lipids, mutations in mtDNA, oxidative stress, and alterations in mitochondrial number [5]–[11].

Many viral proteins target the mitochondria and interfere with its functions contributing to pathology of viral diseases [12], [13]. For example, association of hepatitis C virus (HCV) proteins with the mitochondria play an important role in pathogenesis of HCV induced chronic liver diseases and liver cancer. HCV proteins enter the mitochondria causing an increase in mitochondrial Ca^2+^ uptake, reactive oxygen species (ROS) production and mitochondrial permeability transition. As a result, intrinsic cell death and changes in the liver microenvironment lead to cell transformation [14], [15]. One major factor in HIV pathogenesis is viral protein R (Vpr). Vpr is integrated in the mitochondrial outer membrane and it also reduces the expression of mitofusin 2, which leads to mitochondrial fragmentation and depolarization of ΔΨm inducing death of infected CD4^+^ T lymphocytes [16]. On the other hand, respiratory syncytial virus (RSV) can cause severe infections as viral non-structural protein 1 (NS1) interferes with mitochondrial antiviral signalling protein inhibiting the interferon production [17]. Immune response is therefore delayed early in an RSV infection giving more time for viral replication.

Viruses can modulate mitochondrial functions for their benefit and they can interfere with signalling networks activating growth pathways to increase metabolic activity [18], [19]. One example is the activation of phosphatidylinositol-3 kinases/AKT (PI3K/AKT) survival pathway by rotaviral non-structural protein 1 (NSP1) in the beginning of infection [20]. Another rotaviral protein, NSP4, is integrated into the mitochondrial membranes causing apoptosis through depolarization of mitochondria and release of cytochrome c [20]. NSP1 counteracts the NSP4 induced apoptosis early in the infection giving time for viral replication. Another survival signalling pathway is mediated through the extracellular regulated kinases 1 and 2 (ERK1/2). ERK1/2 signal cascade activates cytoplasmic and nuclear substrates that promote cell survival, cell division, differentiation and cell motility [21]. Overexpression of ERK1/2 has been reported to inhibit the intrinsic mitochondria dependent apoptotic pathway [22]. As a results of its functions, activation of ERK1/2 signalling has been reported to be important mediator in pathogenesis of number of viruses including echovirus 1 [23], coxsackievirus B3 [24], entrovirus 71 [25], vaccinia virus [26], human cytomegalovirus [27], influenza virus [28] and HIV-1 [29]. During virus infection the significance of ERK1/2 activation is mainly to prevent apoptosis and ensure production of viral progeny.

Parvoviruses are small non-enveloped viruses with linear ssDNA genome [30]. Pathology of parvoviral infection is often directly connected to the cytotoxic nature of infection. Enteritis, myocarditis, hepatitis and reticulocytopenia are consequences of parvovirus induced cell death [31]–[35]. The mechanisms of cell death have been reported to be apoptosis, necrosis and death by cytoskeletal rearrangements [34], [36]–[40]. We have used canine parvovirus (CPV) as a model virus in our studies concerning parvovirus pathology and we have earlier reported that a CPV infection initiates apoptosis [41]. CPV induced apoptosis involves activation of caspases (9 and 3) and dissipation of ΔΨm. Caspases are activated early in the infection, but damage to nuclear DNA and depolarization of ΔΨm appear after beginning of viral replication [41]. Others have reported that expression of CPV NS1 protein induces apoptosis in a p53 and Bcl-2 independent fashion [42].

Studies up to this point have reported the events of viral induced apoptosis from late stages of infections. Our aim here is to examine the role of the mitochondria in parvovirus pathology at very early stages of infection. The direct association of CPV with the mitochondria and the consequence of this immediate interaction could potentially be the initial triggers for intrinsic cell death. Alternatively, the NS1 expression and its subsequent cytopathic effects on the cell could also be the original trigger. With both possibilities, a comparison of viral-mitochondrial involvement of early events with later events is reported. Our results demonstrate a close mitochondrial association of CPV, and damage to the morphology of the mitochondria as well as induction of oxidative stress. Interestingly a biphasic compromise of ΔΨm was observed. More significantly, we report the activation of ERK1/2 signalling in parvovirus infection. Knowledge of exact triggers of intrinsic cell death from viral infections will allow better therapeutic interventions and possible alleviation of pathological consequences.

## Materials and Methods

### Cells and virus

Norden laboratory feline kidney (NLFK) cells [43] (gift from Colin Parrish, Cornell University, Ithaca, N.Y.), permissive to CPV, were grown in Dulbecco's modified Eagle's medium (DMEM; Invitrogen Life Technologies, CA, USA) supplemented with 10% fetal calf serum (PAA Laboratories, Pasching, Austria) and 1% PenStrep (Invitrogen Life Technologies, CA, USA). Canine parvovirus type 2 (gift from Colin Parrish, Cornell University, Ithaca, N.Y., USA) derived from an infectious clone, as previously described [44], was propagated in NLFK cells in 500 cm^2^ cell culture flasks (Nunc, Roskilde, Denmark) for 7 days and then stored at −20°C. Cell debris was removed from 300 ml of virus culture medium by centrifugation and the supernatants were concentrated by ultrafiltration (30 kDa filter, Merck Millipore, Darmstadt, Germany). The virus was pelleted by ultracentrifugation at 173 000× g for 1 h and resuspended in 1 ml of PBS pH 7.4. The suspension was sonicated with low power and extracted with chloroform. Full and empty capsids were separated from the CPV containing aqueous layer by isopycnic centrifugation in 45% cesium chloride gradient. Opalescent bands were collected with a syringe and capsids were pelleted by ultracentrifugation at 245 000× g for 4 h. The pellets were resuspended in 100 µl of PBS. Full CPV capsids were used to infect NLFK cells at m.o.i. 10.

### Immunofluorescence microscopy

To study colocalization of CPV with mitochondria, fluorescence confocal microscopy was used. NLFK cells were grown on coverslips and synchronized with 2 mM Thymidine (Sigma-Aldrich, St. Louis, MO, USA) for 18 h. Cells were rinsed with DMEM and inoculated with CPV (m.o.i. 10) in a small volume for 15 min. Then DMEM was added and cells were incubated for 2–24 h. Mock and CPV infected cells were stained with 300 nM MitoTrackerRed (MTR, Invitrogen Life Technologies, CA, USA) for 30 min at 37°C. Cells were fixed with 4% paraformaldehyde, treated with 0.1% Triton in PBS and CPV capsid proteins were labelled with monoclonal anti-CPV antibody (gift from Colin Parrish, [45]) diluted in Triton solution at concentration of 10 µg/ml. Anti-CPV antibody was visualised with Alexa Fluor 488 conjugated anti-mouse antibody (Invitrogen Life Technologies, CA, USA) at a dilution of 1∶200. To label mitochondria with polyclonal anti-COX IV antibody (Abcam, Cambridge, UK) mock and CPV infected NLFK cells grown on coverslips were fixed with ice cold methanol and double labelled with monoclonal mouse anti-CPV antibody and with anti-COX IV antibody at a concentration of 7 µg/ml. Alexa Fluor 488 conjugated anti-mouse antibody and Alexa 594 conjugated anti-rabbit antibody (dilution 1∶200) were used to detect primary antibodies. The samples were examined with a laser scanning fluorescence microscope (LSM 510, Axiovert 100 M; Zeiss, Jena Germany) by using the excitation and emission settings appropriate for the dye used. Single confocal sections were taken from the middle of the cell.

### Colocalization analysis

To quantify the level of colocalization, 10 cells per time point from three independent experiments, 30 cells per time point all together, were randomly selected and imaged using confocal microscope. Levels for the laser power and detector amplification were optimized for each channel in the confocal microscope before starting the quantification. The nucleus was excluded from the image for colocalization analysis. Quantification of colocalization was determined with BioImageXD software [46]. Overlap between channels was expressed as percentage Ch2 voxels colocalizing with Ch1 voxels. The colocalization thresholds were set manually to eliminate background fluorescence and all connected regions with fewer than three pixels were removed to eliminate photon shot noise.

### Immunoelectron microscopy

Cells were grown on 8.8 cm^2^ plastic culture dishes (Nunc, Roskilde, Denmark) to 80% confluency. The cells were synchronized and infected as stated above. After infection dishes were washed with 0.1 M phosphate buffer, pH 7.4 and the cells were fixed with PLP-fixative (0.01 M sodium periodate, 0.075 M lysine, 2% PFA) for 2 h at RT and left in 2% PFA overnight. After rinsing with sodium phosphate buffer, cells were permeabilized for 8 min at RT with phosphate buffer containing 0.01% saponin and 0.1% BSA. Primary antibody (anti-CPV antibody, used at concentration 10 µg/ml) and gold-conjugated secondary antibody (dilution 1∶50; British Biocell International, Cardiff, UK) were diluted in permeabilization buffer and incubated with cells for 1 h at RT. Between and after labelings, cells were washed with permeabilization buffer. Cells were postfixed with 1% glutaraldehyde in 0.1 M phosphate buffer for 10 min at RT, quenched with 50 mM NH_4_Cl in phosphate buffer, and washed with both phosphate buffer and water. Cells were treated in the dark with HQ-silver (British Biocell International, Cardiff, UK) for 2 min, followed by washes with water and gold toning solutions [2% sodium acetate 3×5 min, 0.05% gold chloride (Sigma-Aldrich, St. Louis, MO, USA) 10 min on ice, 0.3% sodium thiosulphate 2×10 min on ice]. After washes with water, the cells were postfixed with 1% osmium tetroxide in 0.1 M phosphate buffer for 1 h at 4°C, dehydrated with a descending concentration series of ethanol, and stained with 2% uranyl acetate. Plastic capsules filled with Epon LX-112 (Ladd Research Industries, Williston, VT, USA) were placed upside-down on top of the cells. Epon was polymerized for 24 h at 45°C and 24 h at 60°C. After polymerization, the capsules were warmed up to 100°C and removed carefully. Horizontal sections were cut, picked up on a grid, and viewed with an electron microscope JEM-1400 (Jeol, Tokyo, Japan). Images were taken with iTEM software (Olympus, Münster, Germany). To analyze the impact of CPV infection on mitochondria, all mitochondria from one cell were classified as intact or damaged and counted. All together mitochondria from 30 cells per time point from three independent experiments were counted.

### Flow cytometric analyses of membrane integrity

To analyse if a CPV infection has an effect on ΔΨm at early time points p.i., JC-1 (Invitrogen Life Technologies, CA, USA) was used. JC-1 is a fluorescent dye that is used to detect the loss of ΔΨm. In healthy cells the dye monomers aggregate and mitochondria can be seen as red, but when mitochondria are depolarized the dye is in a monomeric form and emits green fluorescence. NLFK cells grown in 8.8 cm^2^ dishes (Nunc, Roskilde, Denmark) were synchronized infected (or mock infected) with CPV and incubated for 2–18 h. JC-1 was added to trypsinized cells at a final concentration of 15 µM and incubated for 30 min at 37°C. As a control cells were also labelled in the presence of 100 nM K^+^-selective ionophore valinomycin (Sigma-Aldrich, St. Louis, MO, USA) that collapsed ΔΨm. Stained cells were detected with flow cytometry using a FACSCalibur with a 488 nm laser line and Cell Quest software (Becton Dickinson, Franklin Lakes, NJ, USA) and results were further analysed with FlowJo software (Tree Star, Ashland, OR, USA). A total of 10^4^ cells were considered in each assay to create the cytograms. Cells were gated to two populations according to valinomycin treated cells (depolarized mitochondria) and mock infected cells (polarized mitochondria). Amount of cells showing depolarization of ΔΨm were normalized in comparison with the mock infected control, which was given the value 1. These experiments were repeated three times.

### Detection of ROS

The level of intracellular reactive oxygen species (ROS) was determined with DCFDA cellular ROS detection assay kit (Abcam, Cambridge, MA, USA). Synchronized and infected cells were trypsinized and stained with 20 µM DCFDA for 45 min at 37°C. To induce formation of ROS in positive control, cells were incubated for 2 h with 50 µM tert-butyl hydroperoxide (TBHP) or with 0.5 µM staurosporine (STS) for 16 h. Samples were analysed with flow cytometry using a FACSCalibur with a 488 nm laser line and Cell Quest software (Becton Dickinson, Franklin Lakes, NJ, USA). Results were further analysed with FlowJo software (Tree Star, Ashland, OR, USA). A total of 10^4^ cells were considered in each assay to create the cytograms. Cells were gated to two populations: first population corresponded to the production of ROS as compared to TBHP and STS treated cells; and the second population according to mock infected cells. The amount of cells positive for ROS were normalized in comparison with the mock infected control, which was given the value 1. These experiments were repeated three times.

### Release of calcium

To study the release of calcium from intracellular stores during CPV infection, Fluo-4 Direct calcium assay kit was used (Invitrogen Life Technologies, CA, USA). Measurements were done according to manufacture's instructions. Shortly, cells grown on 96-well plates were synchronized and loaded with Fluo-4 for 60 min at 37°C. Loading solution was changed to DMEM and CPV or 100 nM thapsigargin (Invitrogen Life Technologies, CA, USA), an intracellular calcium releaser, were added to appropriate wells. Fluorescence intensity was measured with Victor X4 2030 Multilabel Reader (PerkinElmer, Waltham, MA, USA) at 30 min – 6 h after treatments using excitation wavelength 485 nm and emission was measured at 535 nm. Fluorescence intensity measurements were normalized in comparison with the mock infected control which was given the value 1. Experiment was repeated three times.

### Activation of cell survival signalling

In order to examine the activation of cell survival signalling in CPV infected cells, phosphorylation of ERK1/2 was studied. NLFK cells were grown and infected with CPV as described above. As a positive control cells were incubated in the presence of 2 µM phorbol 12-myristate 13-acetate (PMA; Sigma-Aldrich, St. Louis, MO, USA) for 1 h (repeated twice). At indicated time points post infection cells were lysed in a 50 mM Tris-HCl (pH 7.4) buffer containing 150 mM NaCl, 1 mM EDTA, 1% NP-40, 0.25% sodium deoxycholate and 1∶100 diluted protease inhibitor cocktail (Sigma-Aldrich, St. Louis, MO, USA). After centrifugation at 14 000× g and 4°C, protein concentration was determined with Bradford method (Bio Rad, Hercules, CA, USA). An amount of 50 µg of proteins were separated on SDS-PAGE gel and transferred to nitrocellulose membrane (Schleicher & Schuell BioScience, Keene, NH). Blots were incubated with polyclonal antibody towards ERK1/2 or phosphorylated ERK1/2 (Cell Signaling Technology, Danvers, MA, USA) at a dilution 1∶1000. Detection was performed with HRP-conjugated goat anti-rabbit antibody (Dako, Glostrup, Denmark) and SuperSignal West Pico Chemiluminescent Substrate (Pierce, Rockford, IL, USA). Densitometry (ImageJ software, NIH, Bethesda, MD, USA) was utilized to measure the intensity of bands. Results were normalized to give a value of 1 for mock infected cells and CPV infected samples were compared to that. Experiments were repeated three times and results are shown as ratio of p-ERK to ERK.

### Inhibition of ERK1/2 activation

To explore the importance of ERK1/2 activation in CPV infection, percentage of infected cells and depolarization of mitochondria were determined in the presence of the MEK1/2 specific inhibitor U0126 (Promega, Madison, WI, USA). U0126 was freshly dissolved at 10 mM in DMSO and added to the culture medium at final concentration of 20 µM for 30 min prior to virus infection and kept in the medium throughout the experiment. Samples were prepared for immunofluorescence microscopy to determine the infection percentage, for immunoblot analysis of ERK1/2 activation and for ΔΨm analysis. Methods for immunofluorescence microscopy, immunoblot and ΔΨm analysis were as previously described. Infection percentage was calculated at 24 h p.i.. Experiments with inhibitor were repeated three times.

### Statistical testing

Student's t­test was used to identify statistically significant differences between mock and CPV infected samples. p≤0.05 was considered significant.

## Results

### CPV colocalized with mitochondria

**Figure 1:**
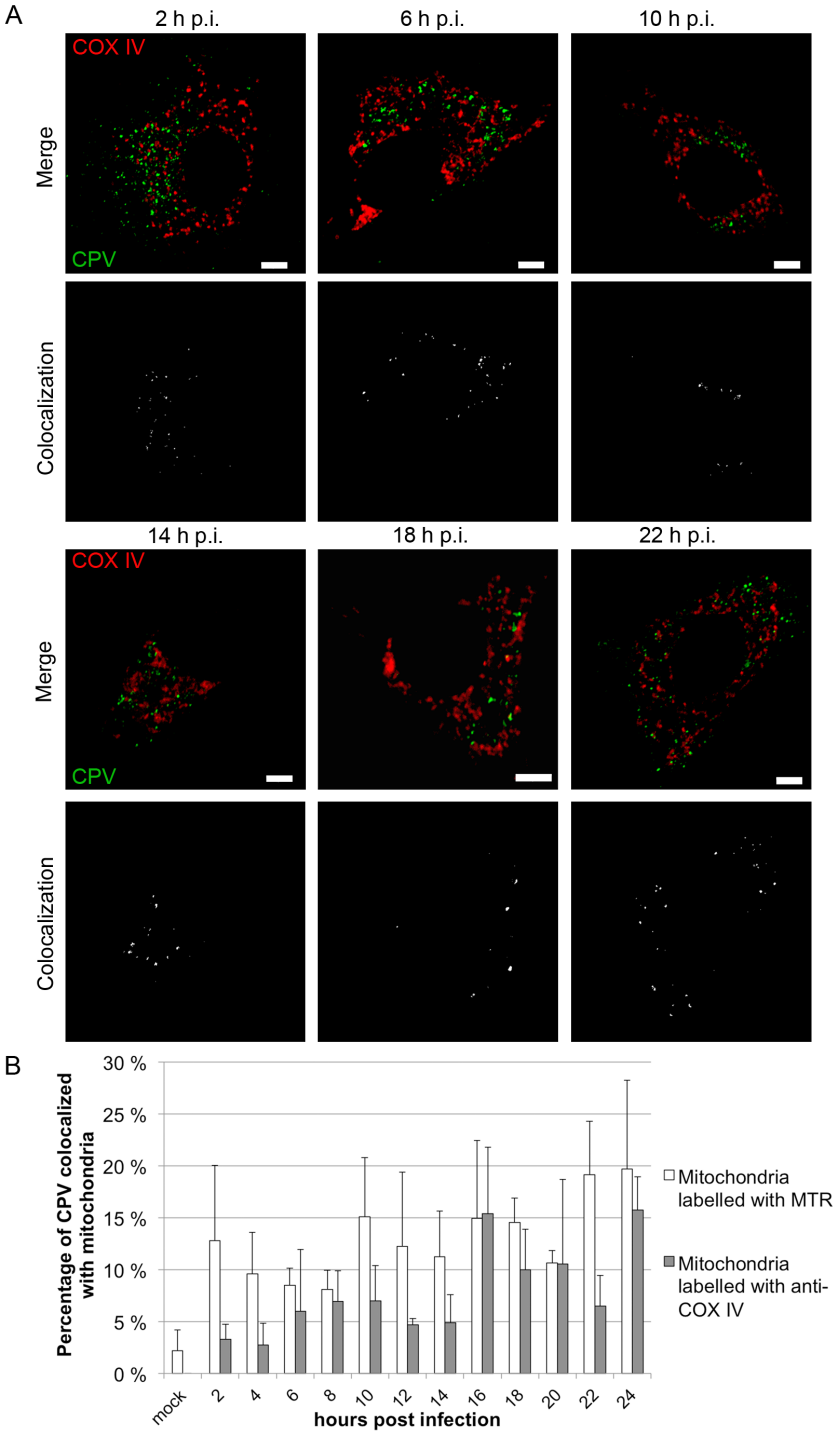
CPV colocalizes with mitochondria. (A) Confocal immunofluorescence images of CPV infected cells. Mitochondria were labelled with anti-COX IV antibody (red) and CPV with anti-capsid antibody (green). Nucleus has been cut out from the images due to really intense fluorescence from CPV at longer time points. Colocalization of CPV with mitochondria is shown in white in lower images. Bars 5 µm. (B) Percentage of colocalization of CPV with mitochondria. Colocalization analysis of confocal microscopy images was done with BioimageXD software. Mitochondria were labelled either with anti-COX IV antibody or with MitoTrackerRed (MTR). CPV was labelled with antibody recognizing intact capsids. Mock 24 h p.i. Results are shown as means from 30 cells from 3 independent experiments ± S.D.

To study the association between CPV and mitochondria, colocalization analysis based on confocal microscopy images was conducted. Results confirmed that cytoplasmic CPV colocalized with mitochondria from 2 h p.i. to 22 h p.i. (Fig. 1A). Colocalization of CPV and mitochondria, as manifested by white merge spots distributed in the cytoplasm, was observed at all time points used (Fig. 1A). Spots had an apparent perinuclear location at 10–14 h p.i..

A quantitative estimation of colocalization was obtained with BioImage XD software and is shown as a mean of 30 individual cells per time point (Fig. 1B). Percentage of cytoplasmic CPV colocalizing (as defined by BioImage XD software) with mitochondria fluctuated during used time points. In MTR labelled cells the colocalization percentage was already 13% at 2 h p.i.. Then colocalization decreased to 9% at 4–8 h p.i.. At 10 h p.i. colocalization increased again to 15% and reached the highest level at 22–24 h p.i. with 19%. With anti-COX IV labelled cells colocalization was low during entry (3–6% at 2–4 h p.i.), but percentage rose after 16 h p.i. and reached a highest level at 24 h p.i. (16%).

### Infection with CPV caused mitochondrial damage

**Figure 2:**
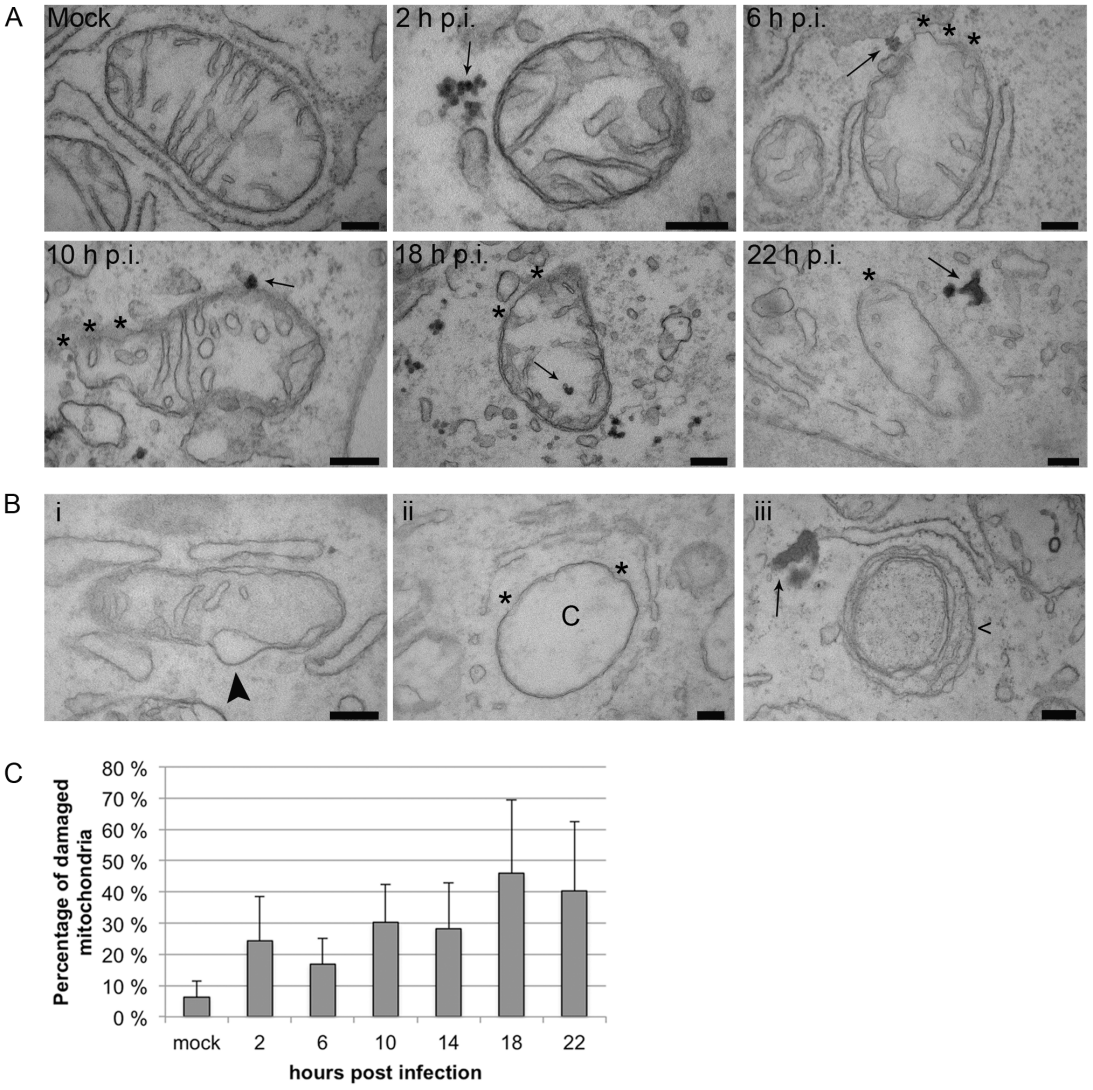
CPV infection influences the morphology of mitochondria. (A) Transmission electron microscopy pictures from mock and CPV infected cells at different time points post infection showing immunogold-labelled CPV to associate with mitochondria. (B) Following a CPV infection damage to the mitochondria can be observed. Images are from 22 h p.i. Infection leads to (i) membrane blebbing (arrowhead), (ii) damage to the membrane (*) and disappearance of cristae (C). (iii) Damaged mitochondria were seen inside autophagosome like structures. C) Percentage of damaged mitochondria was counted with transmission electron microscope from 30 cells from 3 independent experiments. Mock 2 and 22 h p.i. Results are shown as mean ± S.D and there is a statistically significant (p≤0.05) change between mock and CPV infected samples at all time points post infection. Bars 200 nm.

Immunoelectron microscopy was used to detect direct association of CPV with mitochondria and to study the ultrastructure of mitochondria in mock and CPV infected cells (Fig. 2). The preparation of samples within this technique is harsh. Unfortunately this can lead to some loss of the integrity of mitochondria as seen in the percentage of damaged mitochondria in the mock infected sample (6%). CPV located close to mitochondria at all of the studied time points (Fig. 2A). At 22 h p.i. virus label was seen in large plaques, not as rounded gold particles. These plaques were not located on the mitochondrial membrane as some label with the earlier time points. Some gold label was also seen occasionally inside the mitochondria at all time points. Damage to the mitochondrial morphology was seen in CPV infected cells. Infection seemed to cause disintegration of the membrane (as in Fig. 2A 6, 10, 18, 22 h p.i., 2B ii; marked with *). Additionally, mitochondrial membrane blebbing (Fig. 2B i, arrowhead) and disappearance of cristae (Fig. 2B ii, marked with C) was seen. At later time points starting 14 h p.i. damaged mitochondria were seen in autophagosome-like structures (Fig. 2B iii, marked with <). Percentage of damaged mitochondria was counted from 30 cells at each time point used and the mean is shown in Figure 2C. At 2 h p.i. the percentage of damaged mitochondria was 24%, but it decreased to 17% by 6 h p.i.. After this, the percentage rose and was the highest at 18 h p.i. when the percentage of damaged mitochondria reached 46%. These results demonstrated that CPV infection induced damage to the mitochondria.

### Depolarization of mitochondrial membranes was associated with CPV infection

**Figure 3:**
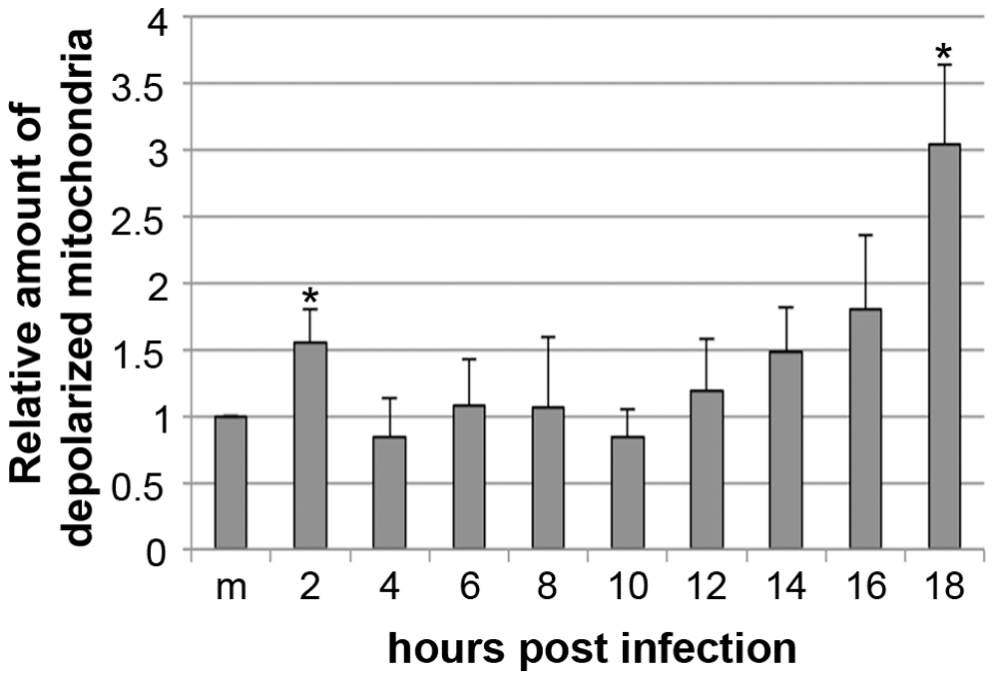
Biphasic loss of mitochondrial membrane potential during CPV infection. Flow cytometry of JC-1 labelled cells was used to detect depolarization of mitochondrial membrane potential during CPV infection. Results are shown as mean of relative amount of cells showing mitochondrial depolarization from three independent repeats (m, mock infected 18 h p.i.). * The difference is statistically significant (p≤0.05) between marked time point and mock infected cells.

JC-1 dye was used to study the potential of mitochondrial membrane. CPV infected cells were labelled with this dye at different times post infection. Results demonstrated that mitochondria began to be depolarized at 2 h p.i. (Fig. 3) when the amount of depolarized mitochondria was 1.6 times the amount in mock infected cells. At 4 h p.i. ΔΨm was restored to the level of mock infected cells. However, at 14 h p.i. depolarization of the mitochondrial membranes was seen again. At 18 h p.i. the amount of depolarized mitochondria was tripled when compared to mock infected cells. Valinomycin was used to induce depolarization of ΔΨm in positive controls, and with this drug there were 27±4.1 (p≤0.05) times more depolarized mitochondria than in mock infected cells (data not shown). These results indicate that mitochondria are depolarized during CPV infection.

### The ROS level increases during the early phases of CPV infection

**Figure 4:**
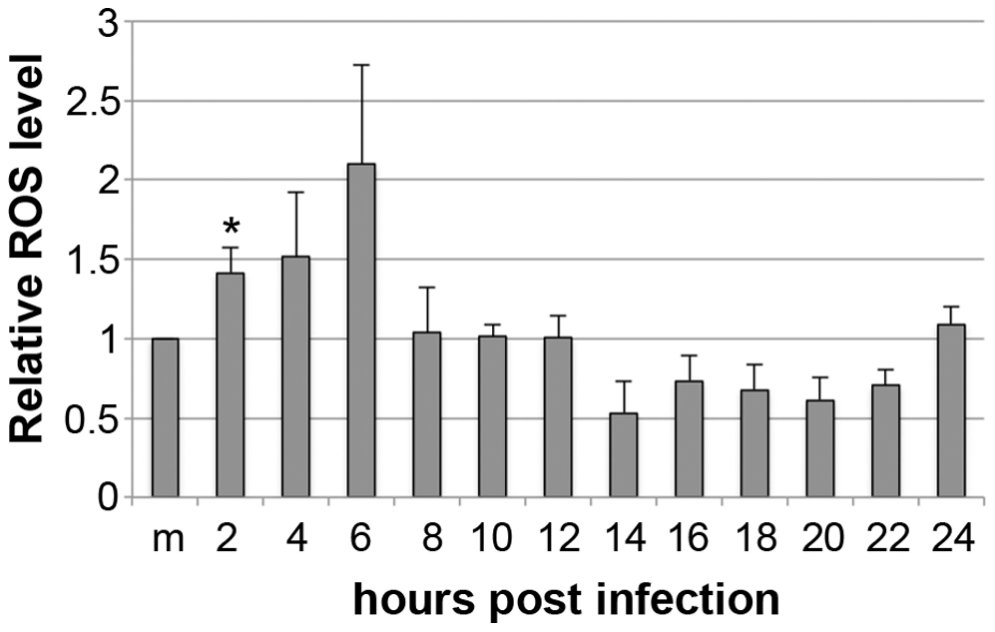
Production of ROS during CPV infection. The level of ROS was determined by loading the cells with DCFDA and analyzed by flow cytometry. Results are shown as mean of relative amount of cells showing production of ROS from three independent repeats (m, mock infected 24 h p.i.). * The difference is statistically significant (p≤0.05) between marked time point and mock infected cells.

Production of ROS during CPV infection was analysed with DCFDA. The intracellular level of ROS increased in the beginning of infection (Fig. 4). The amount of cells with increased ROS level at 2 h p.i. was 1.4 times the level in mock infected cells. Production of ROS continued to increase up to 6 h p.i., but returns to the level of mock infected cell from 8 h–22 hr p.i.. Thereafter the level of ROS increased again at 24 h p.i.. In STS treated apoptotic cells the level of ROS was 6.4±1.5 (p≤0.05) times the level in mock infected cells and in TBHP treated cells the factor was 6.0±0.4 (p≤0.05) (data not shown). These results demonstrate that mitochondria are affected during virus uptake, but cells are able to recover from the triggered oxidative stress.

### Cytoplasmic calcium concentration remained unchanged in the beginning of infection

**Figure 5:**
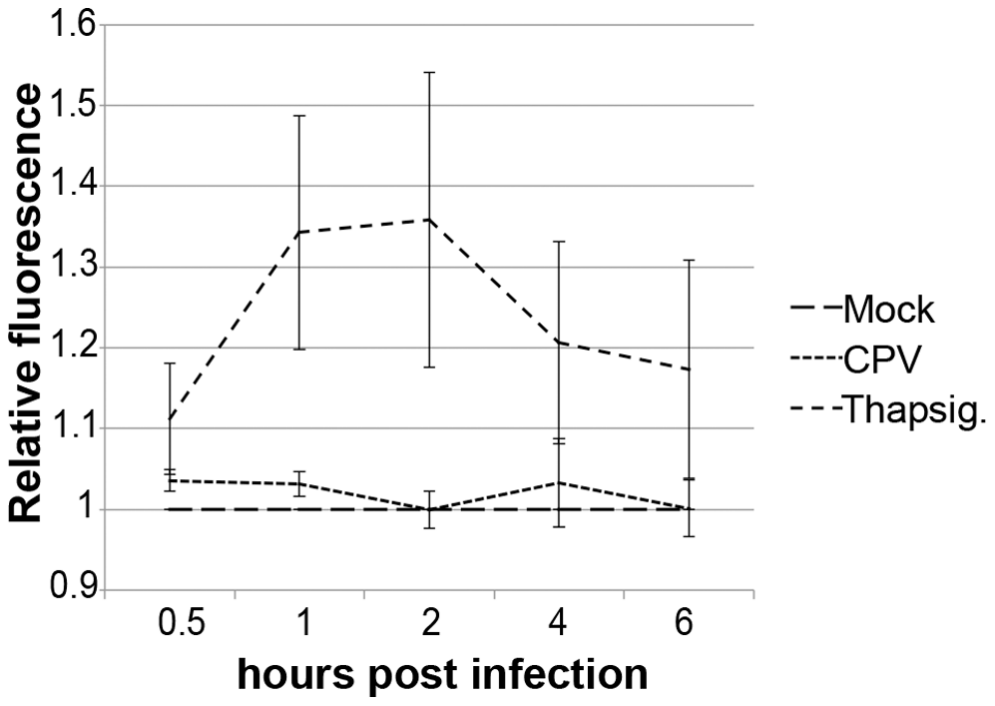
Calcium is not released to the cytoplasm during the entry phase of infection. Release of calcium from intracellular stores, like the mitochondria, was studied with Fluo-4 dye. Cells were loaded with Fluo-4 and infected with CPV. At indicated time points p.i. fluorescence intensity was measured. Thapsigargin was used as a positive control. Results are means from 3 repeat ± S.D.

Fluorescent calcium indicator Fluo-4 was used to detect changes in cytoplasmic calcium concentration. Cells were loaded with Fluo-4 and treated with CPV or thapsigargin. Thapsigargin releases calcium from intracellular stores by inhibiting calcium-ATPases on endoplasmic reticulum. CPV infected and thapsigargin treated cells were compared to mock infected cells. Thapsigargin induced direct increase in cytoplasmic calcium when added to the cells as detected by increase in fluorescence (Fig. 5). In CPV infected cells only a minor change was observed in the fluorescence level indicating small fluctuation in cytoplasmic calcium concentration at 0.5–6 h p.i when compared to mock infected cells.

### ERK1/2 was activated during a CPV infection

**Figure 6:**
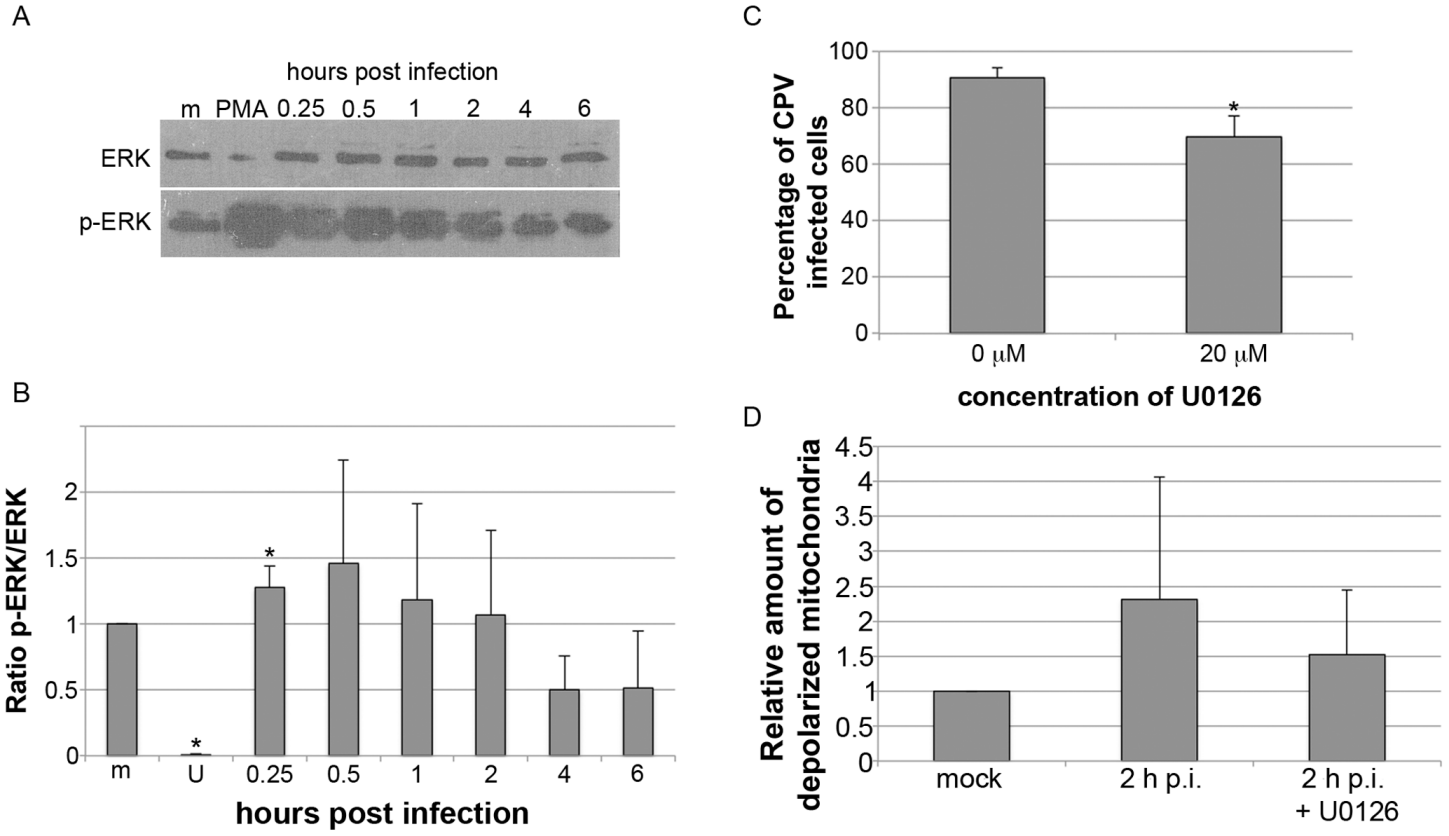
ERK1/2 signalling is activated early in CPV infection. (A) Activation of ERK1/2 was analyzed based on phosphorylation of ERK1/2 (p-ERK1/2). Cell lysates were collected at indicated time points post infection and immunoblotting with antibody towards ERK or p-ERK1/2 was performed. (B) Intensity of band signals were determined with densitometry (ImageJ software) and ratio of p-ERK to ERK was calculated. * The difference is statistically significant (p≤0.05) between marked time point and mock infected cells (m, mock infected 6 h p.i.; U, U0126). (C) CPV infection was carried out in the presence of 20 µM U0126 that inhibits the activation of ERK1/2. At 24 h p.i. cells were fixed and CPV infected cells were visualized with anti-capsid antibody. Infection percentage was determined with immunofluorescence microscopy. The observed decrease in infection percentage was statistically significant (p≤0.05). (D) Cells were infected with CPV or mock infected with or without 20 µM U0126 and JC-1 was used to detect the depolarization of mitochondrial membrane potential. Results are shown as mean of relative amount of cells showing mitochondrial depolarization from three repeats.

Due to the discovered changes in ΔΨm at 2–4 h p.i., activation of cell survival signalling in the beginning of infection was studied. We used antibody towards phosphorylated ERK1/2 (p-ERK1/2) to investigate activation of ERK1/2 signalling. A representative result is shown in Fig. 6A. Ratio of band intensities of p-ERK1/2 to ERK1/2 increased already at 15 min p.i. (Fig. 6B). Activation was further enhanced to 30 min p.i.. Thereafter the activation started gradually to decline and by 4 h p.i. the ratio was below that of mock infected cells indicating a decrease in p-ERK. For cells incubated with PMA the ERK ratio was 9.8±0.6 (p≤0.05) (data not shown). When the activation of ERK1/2 was inhibited by U0126 the percentage of infection declined 20% when compared to infection without the drug (Fig. 6C). The used concentration of U0126 inhibited the activation of ERK1/2 efficiently as detected with immunoblotting (Fig. 6B). Inhibiting the ERK1/2 activation reduced the CPV induced depolarization of ΔΨm at 2 h p.i. (Fig. 6D).

## Discussion

Taking into account the important role of mitochondria in producing energy, defects in the mitochondrial functions reflect directly to the viability of the cell. Indeed, mitochondrial dysfunction is linked to pathologies of diseases among viral diseases [8], [9], [13], [47]. As viruses are able to modulate mitochondrial function for their benefit this feature has evoked interest to be targeted for antiviral drugs. New antiviral drugs could inhibit viral changes in mitochondrial function or induce proapoptotic effect in virus-infected cells to inhibit viral replication [13], [18], [48]. To be able to design new antiviral drugs and to develop better intervention strategies, various virus-mitochondria interactions have to be carefully studied.

We have analysed the role of mitochondria in parvovirus pathology. Our data demonstrates that CPV localized to the cytoplasm near mitochondria at 2–24 h p.i. (Fig. 1). CPV associated with mitochondria through out the infection, but was only occasionally seen inside the mitochondria (Fig. 1 and 2). The percentage of CPV colocalized with mitochondria showed that at 2–24 h p.i. a fraction of CPV is associated with the mitochondria (Fig. 1). The amount of viruses associated with the mitochondria increased with longer time points after 16 h p.i.. By this time new viruses have been made [41], [49] and the virus content of the cell has increased [41] probably affecting also the amount of association of CPV with the mitochondria. Colocalization percentages vary between the two used mitochondria label (Fig. 1B); MTR goes in the mitochondria, and labels the whole cell organelle as COX IV localizes at inner mitochondrial membrane. Different labelling of the mitochondria with these dyes could account for the differences seen in colocalization percentages (Fig. 1B). Electron microscopy experiments showed that CPV was mainly associating with outer mitochondrial membrane (Fig. 2A) and not with inner mitochondrial membrane containing COX IV. Some of the increased mitochondrial damaged seen in the EM micrographs compared to the loss of membrane potential (Fig. 3) may also be due to the harsh EM technique. However as can be seen in Fig. 6 there is an activation of ERK that could counteract the loss of membrane potential at early stages of infection making this loss not a feature of lethal mitochondrial damage. To our knowledge, parvoviral proteins have not been earlier reported to associate with the mitochondria. However, localization of viral proteins in mitochondria has been reported for example for HCV [14], RSV [17], rotavirus [20], HIV [16] and herpes simplex virus [50].

During HCV infection, viral proteins target the mitochondria and cause enlargement of mitochondria and disappearance of cristae [14]. Vpr and gp160 proteins of HIV have been reported to induce deformation of cristae and disappearance of outer membrane [16], [51]. EM studies presented that CPV infection induced similar morphological changes to the mitochondria (Fig. 2). These include rupture of mitochondrial membrane, disappearance of cristae and membrane blebbing. Damaged mitochondria started to collect after 10 h p.i.. Incoming virus (0–10 h p.i.) affected the mitochondrial structure only transiently, but expression of NS1 (after 10 h p.i. [41], [49]) and production of new viruses further damaged the mitochondrial structure. Figure 2C shows less damage to the mitochondria at 22 h p.i. than at 18 h p.i.. This decline depends on the decreased cell amount as infected cells start to die and detach from the dish at 22 h p.i.. Incubation of purified mitochondria from HeLa cells with a parvovirus minute virus of mice (MVM) did not induce damage to mitochondrial membranes [52]. Furthermore, microinjected MVM did not damage mitochondria in *Xenopus* oocytes although MVM was able to rupture nuclear envelope [52]. The reported results differ from our results in that we demonstrate that CPV damages the mitochondria during an infection. However, in experiments with MVM [52] there was no viral replication and in our experiments the damaged mitochondria accumulated after the beginning of viral replication (Fig. 2C).

Autophagocytosis is a normal way for cells to remove damaged and malfunctioning mitochondria to favour cell surviving [53], [54]. Double membrane vesicle is a characteristic of autophagosomes [55], [56]. EM studies revealed that damaged mitochondria were seen inside structures resembling autophagosomes (Fig. 2B, iii) at later time points, starting from 14 h p.i.. Infection of UT7/Epo-S1 cells with human parvovirus B19 (B19V) has been reported to induce autophagocytosis [57]. In B19V infection autophagosomes contained degraded mitochondria and the autophagocytosis was connected to the survival of infected cells from apoptosis. Similarly, in our studies the autophagosome-like structures contained damaged mitochondria. However, the implication of observed autophagocytosis remains to be elucidated.

The mitochondrial function is dependent on intact mitochondria. One indicator of mitochondrial health is ΔΨm the dissipation of which is connected to the intrinsic mitochondrial apoptotic pathway [58]. Our experiments revealed that mitochondria were depolarized shortly in the beginning of infection (Fig. 3). However, the final depolarization of larger population of mitochondria appeared later, about 14–16 h p.i. (Fig. 3), by the time that viral replication has started as detected by the NS1 expression [41]. Dissipation of ΔΨm indicates permeabilization of mitochondrial inner membrane that leads to cell death [3], [59], [60]. AAV-2, belonging to the adeno-associated parvoviruses, has been reported to sensitize cells to apoptosis by targeting mitochondria [61]. In combination with cisplatin AAV-2 induced depolarization of ΔΨm that was not detected with AAV-2 or cisplatin alone. However, the mechanism involved in sensitizing mitochondria has not been studied. Increased production of ROS is often associated with mitochondrial, proteins, lipids and DNA damage [62]. Additionally ROS play a role in induction of cell death as reported for rat parvovirus H-1 [63]. The level of ROS increased in our studies during the early phases of infection (Fig. 4). However, at 8 h p.i. the level of ROS had declined to the level of mock infected cells indicating that the production of ROS only at later time points (post-24 h) could be a contributing element to apoptosis. The final factor to induce cell death could also be mediated through a synergy of other pathways, such as DNA damage and caspase activation [41], [64], [65]. B19V induces apoptosis through mitochondrial cell death pathway in non-permissive cells [66]. The trigger for the intrinsic apoptotic route is from NS1 induced DNA damage [64], [65]. Our results suggest that mitochondrial pathway is involved in CPV induced cell death, possibly by a mechanism similar to that observed for B19V [65]. The direct contact observed between CPV and mitochondria in the beginning of infection (Fig. 1 and 2, 0–10 h p.i.) did not launch an apoptotic process, but was triggered after initiation of viral replication as detected by NS1 expression [41].

Parvoviruses harbour a phospholipase A2 (PLA2) activity in their VP1 protein [67]. Expression of B19V VP1 protein has been reported to increase the cytoplasmic calcium concentration by activating I_CRAC_ channels on plasma membrane in endothelial cells [68]. Additionally, cytoplasmic calcium concentration controls ΔΨm [1] and infection of viruses, involving poliovirus, rotavirus, hepatitis B virus and HIV, induces an increase in cytoplasmic calcium concentration that is connected to the depolarization of ΔΨm and initiation of apoptosis [69]–[72]. Due to the observed change in ΔΨm at 2 h p.i. (Fig. 3) we analysed the changes of the intracellular calcium concentration. However, the calcium concentration was stationary (Fig. 5).

When the colocalization of CPV with mitochondria was studied by labelling mitochondria with MTR the percentage of colocalization was already 13% at 2 h p.i. (Fig. 1). At the same time damage to mitochondria and change in the mitochondrial homeostasis could be seen as some mitochondria lost their ΔΨm and the level of intracellular ROS increased (Fig. 2C, 3 and 4). The mitochondria can sense intracellular stress through different signalling cascades and responds to those in order to get back to homeostasis [3]. In CPV infected cells the homeostasis was gained back at 4 h p.i. as the ΔΨm was normalized to the level of mock infected cells (Fig. 3). As ERK1/2 signalling is involved in inhibition of apoptosis at the mitochondrial level [22] we tested for ERK1/2 activation, a cell survival signal, in the very early phases of a CPV infection (Fig. 6). It is demonstrated that indeed ERK1/2 was activated, as could be detected with an increase in p-ERK (Fig. 6A, B), at 15 min – 2 h p.i.. Additionally, inhibition of ERK1/2 activation partially prevented depolarization of ΔΨm at 2 h p.i. (Fig. 6D). ERK1/2 activated signalling promotes cell survival and prevents the stress situation evoked by the cell due to invading viruses. A virus benefits from this kind of signalling by receiving more time for viral replication as seen with other viruses like coxsackievirus B3, enterovirus 71 and influenza virus [24], [25], [28]. In parvovirus studies, inhibition of ERK1/2 activation by U0126 decreased the ability of CPV to replicate in host cells (Fig. 6C) indicating the importance of ERK1/2 activation. In another study, stimulation of macrophages with B19V VP1 protein, that contains PLA2 domain, has been reported to also activate ERK1/2 signalling [73]. In addition, activation of ERK1/2 signalling decreased during a B19V infection in CD36^+^ erythroid progenitor cells (EPC) cultured under hypoxia [74]. The B19V was shown to replicate more efficiently under hypoxia and in these culture conditions one important factor favourable for B19V replication was the decrease of ERK1/2 activation. In CD36^+^ EPCs the decline in activated ERK1/2 was connected to the lower level of EPC differentiation [74]. Here we report reverse action of ERK1/2 signalling during parvovirus infection. However, for both CPV and B19V the change in ERK signalling accounts for the purpose of ensuring the production of progeny. Other survival pathways, like the PI3K/AKT pathway utilized by rotavirus [20] or pathways inhibiting apoptosis [75], are possibly having a role during CPV infection.

Parvoviral NS1 protein is known for its cytotoxic nature [35], [36], [42], [63]–[65]. Changes of the mitochondria displayed in this study occurred concomitantly with the earlier reported schedule of NS1 expression [41], [49]. We have earlier shown that caspase 9, the caspase involved in intrinsic apoptotic pathway, is activated already 6 h p.i.. Significant activation of caspase 3 and damage to nuclear DNA can be detected at 12 h p.i. [41]. By 6 h p.i. viral replication has not started yet, consequently this activation is likely to happen through cellular signalling pathways. This is supported by the results of this study as mitochondrial structures were mainly intact at 6 h p.i. (Fig. 2 and 3). Increase in damaged mitochondria (Fig. 2 and 3) and DNA [41] could be seen after time of onset of viral protein expression, especially that of NS1, after 12 h p.i.. Even though CPV was shown to associate with the mitochondria directly, this contact does not seem to induce cell death. Early damage to mitochondria at 2 h p.i. (Fig. 3), production of ROS (Fig. 4) and caspase activation at 6 h p.i. [41] indicate that virus infection is sensed by the host and it reacts not by being pushed to intrinsic cell death but to cell survival. As seen in Fig. 3, the mitochondrial membrane potentials are of the same levels as with mock infected cell between 4–12 h p.i.. This may be due to the long lasting effect of the activated ERK signalling cascade. We speculate that the activated ERK1/2 signalling is involved in regaining the mitochondrial homeostasis along with other survival signalling pathways and apoptotic changes are starting to collect only after 14 h p.i. (Fig. 3), after beginning of viral replication and NS1 expression. Taken together, there is association of CPV and the mitochondria at very early time points and onwards, but this is not the initial signal to push the cell into apoptosis. ERK1/2 signalling is activated in the beginning of infection to ensure cellular viability. After beginning of viral replication the involvement of mitochondria in the cell death can be seen as indicated by mitochondrial damage (Fig. 2 and 3). Results obtained with this study are useful for understanding parvoviral pathology and also in more general scale provide information about virus-mitochondria association.
